# In-season monotony, strain and acute/chronic workload of perceived exertion, global positioning system running based variables between player positions of a top elite soccer team

**DOI:** 10.1186/s13102-021-00356-3

**Published:** 2021-10-12

**Authors:** Rafael Oliveira, Alexandre Martins, Hadi Nobari, Matilde Nalha, Bruno Mendes, Filipe Manuel Clemente, João Paulo Brito

**Affiliations:** 1 Sports Science School of Rio Maior–Polytechnic Institute of Santarém, 2140-413 Rio Maior, Portugal; 2 Life Quality Research Centre, 2140-413 Rio Maior, Portugal; 3 Research Center in Sport Sciences, Health Sciences and Human Development, 5001-801 Vila Real, Portugal; 4 Department of Exercise Physiology, Faculty of Educational Sciences and Psychology, University of Mohaghegh Ardabili, Ardabil, 56199-11367 Iran; 5 Sepahan Football Club, Isfahan, Iran; 6 Falculty of Human Kinetics, University of Lisboa, Lisbon, Portugal; 7 Escola Superior Desporto e Lazer, Instituto Politécnico de Viana do Castelo, Rua Escola Industrial e Comercial de Nun’Álvares, 4900-347 Viana do Castelo, Portugal; 8 Delegação da Covilhã, Instituto de Telecomunicações, 1049-001 Lisbon, Portugal; 9 Comprehensive Health Research Centre (CHRC), Departamento de Desporto e Saúde, Escola de Saúde e Desenvolvimento Humano, Universidade de Évora, Évora, Portugal

**Keywords:** ACWR, High-speed running, In-season, Positions, RPE, Soccer, Total distance

## Abstract

**Background:**

The interpretation of the load variations across a period seems important to control the weekly progression or variation of the load, or to identify within- micro and mesocycle variations. Thus, the aim of this study was to describe the in-season variations of training monotony, training strain, and acute: chronic workload ratio (ACWR) through session rating of perceived exertion (s-RPE), total distance and high-speed running (HSR) according to playing positions in an elite soccer team.

**Methods:**

Seventeen professional players from an European First League team participated in this study. They were divided four central defenders (CD), three wide defenders (WD), four central midfielders (CM), three wide midfielders (WM) and three strikers (ST). The players were monitored daily over a 41-week period of competition where 52 matches occurred during the 2015–2016 in-season. Through the collection of s-RPE, total distance and HSR, training monotony, training strain and ACWR were calculated for each measure, respectively. Data were analysed across ten mesocycles (M: 1–10).

**Results:**

The main results showed significant differences (*p* < 0.05) for TMs-RPE between CD vs. ST (moderate effect) in M2; between CD vs. CM (moderate effect) for TS of s-RPE; between CD vs. ST moderate effect) in M6 for ACWR of s-RPE. In addition, there was significant difference between CM vs. ST (moderate effect) in M2 for TS of TD; between WD vs. ST (moderate effect) in M3 for ACWR of TD. Moreover, there were significant differences for TM of HSR between CD vs. WD (very large effect); CD vs. WD (moderate effect) in M4 for TS of HSR.

**Conclusions:**

The present study presents new insights to coaches and technical staff about the variation profiling of TM, TS, and ACWR calculated with internal and external load measures, between player positions during 10 mesocycles.

## Introduction

The training load (TL) monitoring is considered as an almost mandatory duty of coaches and practitioners elite soccer teams [1]. It helps to better apply the load in order to maximize players performance in match-days, it could avoid getting injured or negatively fatigued along with a better periodization training process [2–4].

In this regard, there are several measures used to training load (TL) quantification. On one hand measures such as session rating perceived of exertion (s-RPE), heart rate or blood lactate which are related to internal load while, on the other hand, measures such total distance, running speed thresholds, or accelerometry-based variables are related to external load [4]. Together, both internal and external load provided relevant information for coaches, staff, and scientist in order to achieve best results possible [5].

Through the measures presented before, it is possible to produce helpful workload measures that will highlight the variations that occur over the season, specifically the within microcycle or mesocycle variations [6]. Some of the most known methods are training monotony (TM), training strain (TS), and acute: chronic workload ratio (ACWR). For instance, TM represents the load variation within the week while, TS represents the overall stress produced by the load over the week [7]. Finally, ACWR represents the relationship between the load applied for one week and the load applied in the previous 4 weeks [8].

As mentioned, the workload measures presented seem to be sensitive over the season and when analysed with other contextual factor such as player status, player positions or congested periods, it is expected that different contextual factors present distinct values. Previous studies in professional soccer players, it was found that TM of accelerometry-based measures were meaningfully greater in three matches during congested weeks, than those participating in two or one [9]. Similar results were found in comparisons between starters and non-starters regarding the workload measures of *new body load* and metabolic power [10] or accelerometry-based variables [11].

With special regard to player positions, Di Salvo et al. [12] showed that soccer matches displayed significant difference between different player positions in elite soccer teams. In this sense, a recent study found greater TS for wide defenders and wingers with respect to high-speed running (HSR) and number of sprints when compared with the other positions [13]. However, and to the best of our knowledge, this was the only study that analysed such workload measures (without including ACWR) between player positions.

Due to the limited research, especially in elite soccer teams, more evidence is needed to provide detailed descriptions over the in-season to help coaches, staff, and scientific community to better develop training strategies to maximize performance of the player for competitions. Based on that, the purpose of this study was to describe the in-season variations of TM, TS, and ACWR through s-RPE, total distance, and high-speed running (HSR) between player positions of an elite soccer team.

## Material and methods

### Subjects

Seventeen elite soccer players (aged, 25.4 ± 4.1 years) participated in this study. The players belong to a team that participated in UEFA Champions League. The participating players included the following field positions: four central defenders (CD), three wide defenders (WD), four central midfielders (CM), three wide midfielders (WM), and three strikers (ST) [14]. The inclusion criteria were regular participation in most of the training sessions (80 % of weekly training sessions), while the exclusion criteria include lack of player information, illness and/or injury for two consecutive weeks. Goalkeepers were excluded from the study. All participants were familiarized with the training protocols. All players and their parents signed informed consent prior to the investigation. This study was conducted according to the requirements of the Declaration of Helsinki and was approved by the Ethics Committee of Polytechnic Institute of Santarém (252020Desporto).

### Experimental approach to the problem

Training load data were collected over a 41-week period of competition where occurred 52 matches during the 2015–2016 in-season. The team used for data collection competed in four official competitions across the season, including UEFA Champions League, the national league, and two more national cups from their own country. For the purposes of the present study, all the sessions carried out as the main team sessions were considered. This refers to training sessions in which both the starting and non-starting players trained together. Only data from training sessions were considered. Data from rehabilitation or additional training sessions of recuperation were excluded. This study did not influence or alter the training sessions in any way. Training data collection for this study was carried out at the soccer club’s outdoor training pitches of natural grass. Total minutes of training sessions included warm-up, main phase, and slow down phase plus stretching.

The season was organized into 10 mesocycles (M: 1–10) according to previous studies [15–17] and to coaches’ decisions instead of two/three periods of the season that could influence results interpretation. The number of training sessions, number of competitive matches, and total amount of training duration for starters and non-starters is presented in Table 1.

**Table 1:** Number of training sessions, session duration per player position (min) and number of competitive matches during the 41-week period

Mesocycle (M)	M1	M2	M3	M4	M5	M6	M7	M8	M9	M10
Training sessions number	16	20	18	18	20	20	19	20	18	20
Session duration of central defenders (min)	1569	1778	1456	1077	1489	1659	1456	1420	1250	1268
Session duration of wide defenders (min)	1595	1699	1527	1314	1593	2097	1343	1353	1354	1385
Session duration of central midfielders (min)	1623	1725	1426	1196	1596	1839	1524	1382	1311	1425
Session duration of wide midfielders (min)	1574	1729	1518	1126	1547	2153	1492	1372	1356	1368
Session duration of strikers (min)	1603	1769	1509	1241	1595	1832	1546	1343	1378	1441
Number of matches	4	5	4	5	6	8	5	4	7	4

### Internal training load quantification

CR10-point scale, adapted by Foster et al. was applied [18] 30 min after the end of each training session. Players used an app on a tablet to individually provide their RPE value. The scores provided were then multiplied by the training duration, to obtain the s-RPE [18, 19]. The players were previously familiarized with the scale, and all the answers were provided individually to avoid non-valid scores.

### External training load quantification

Global positioning system (GPS) units (Viper pod 2, STATSports, Belfast, UK) with 10-Hz sampling rate were used to monitor training duration, total distance and HSR (above 19 km/h) for each player. For better satellite reception of the GPS antenna, GPS unit was placed on the upper back between the left and right scapula through a custom-made vest. Previously, Beato et al. [20] positively tested the validity and reliability of linear, multidirectional, and soccer-specific activities through this system. Thirty minutes before the start of training session, all devices were turned on to acquire satellite signals and to provide synchronization between the GPS clock and the satellite’s atomic clock. After training sessions, the Viper PSA software (STATSports, Belfast, UK) was used to download data and to clip all training session (i.e., from the beginning of the warm-up to the end of the last organised drill). In order to avoid inter-unit error, players wore the same GPS device in each training sessions.

### Calculations of training workload measures

Through s-RPE, total distance and HSR, the following variables were calculated: (1) TM (mean of training load during the 7 days of the week divided by the standard deviation of the training load of the seven days) [10, 13, 21]; (2) TS (sum of the training loads for all training sessions during a week multiplied by training monotony) [10, 13, 21]; and (3) ACWR (dividing the acute workload, 1-week rolling workload data, by the chronic workload, the rolling 4-week average workload data) [22–24].

### Statistical analysis

Data were analysed using SPSS version 22.0 (SPSS Inc., Chicago, IL) for Windows statistical software package. Initially, descriptive statistics were used to describe and characterize the sample. Shapiro-Wilk and the Levene tests were used to assumption normality and homoscedasticity, respectively. Repeated measures ANOVA was used with Bonferroni post hoc adjustment once variables obtained normal distribution (Shapiro-Wilk > 0.05) and it was used ANOVA Friedman and Mann–Whitney tests for the variables that not obtained normal, to compare different M and groups. Hedge’s g effect size (95 % confidence interval) was also calculated. The Hopkins’ thresholds for effect size statistics were used, as follows: ≤ 0.2, trivial; > 0.2, small; > 0.6, moderate, > 1.2, large, > 2.0, very large and > 4.0, nearly perfect [25]. Results were considered significant with *p* ≤ 0.05.

## Results

**Fig. 1:**
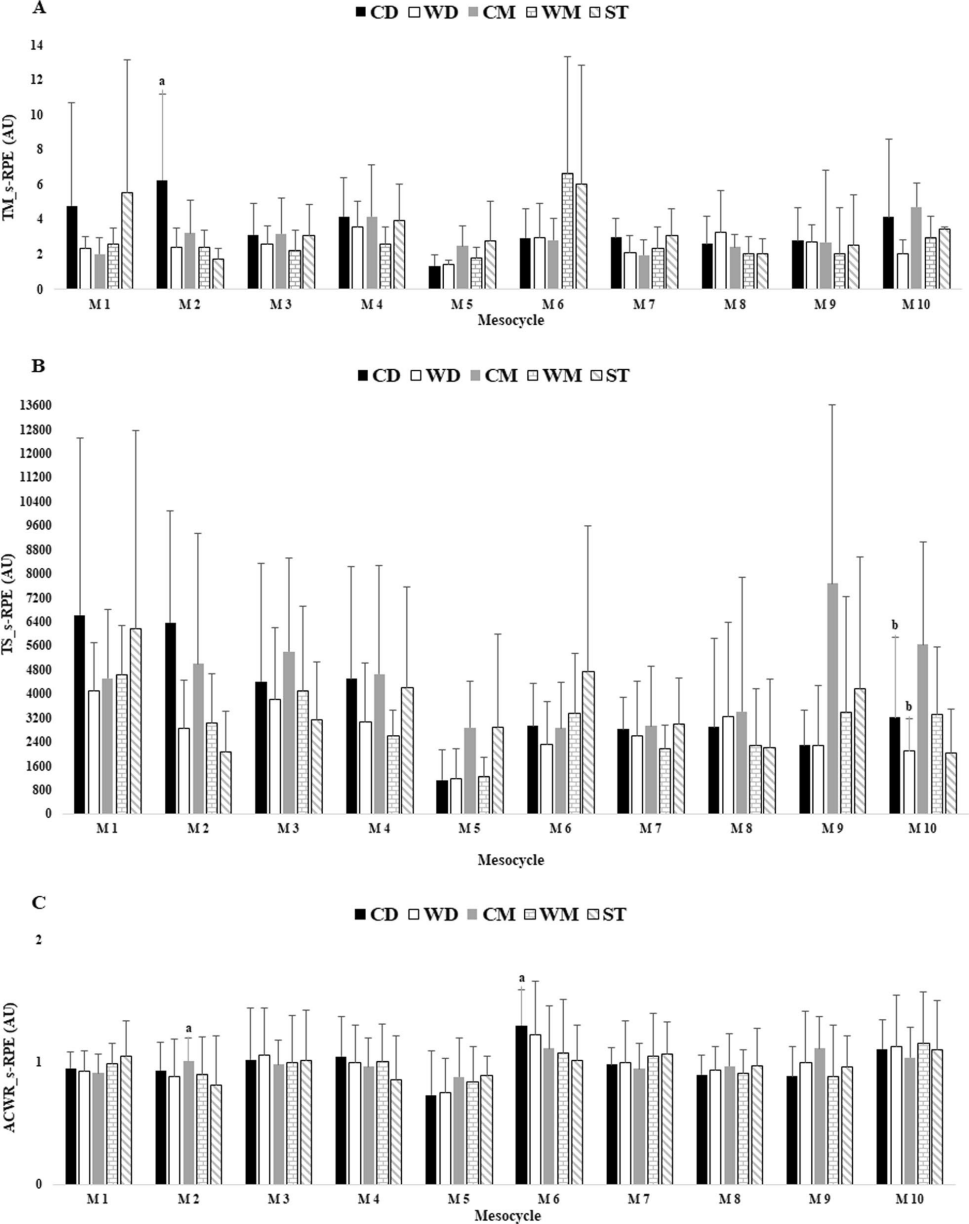
TM, TS and ACWR variations calculated through the s-RPE across 10 mesocycles weeks by player positions. **a** TM_s-RPE; **b** TS_s-RPE; **c** ACWR_s-RPE. M: mesocycle; a: denotes difference from strikers; b: denotes difference from CM. All, p < 0.05

**Fig. 2:**
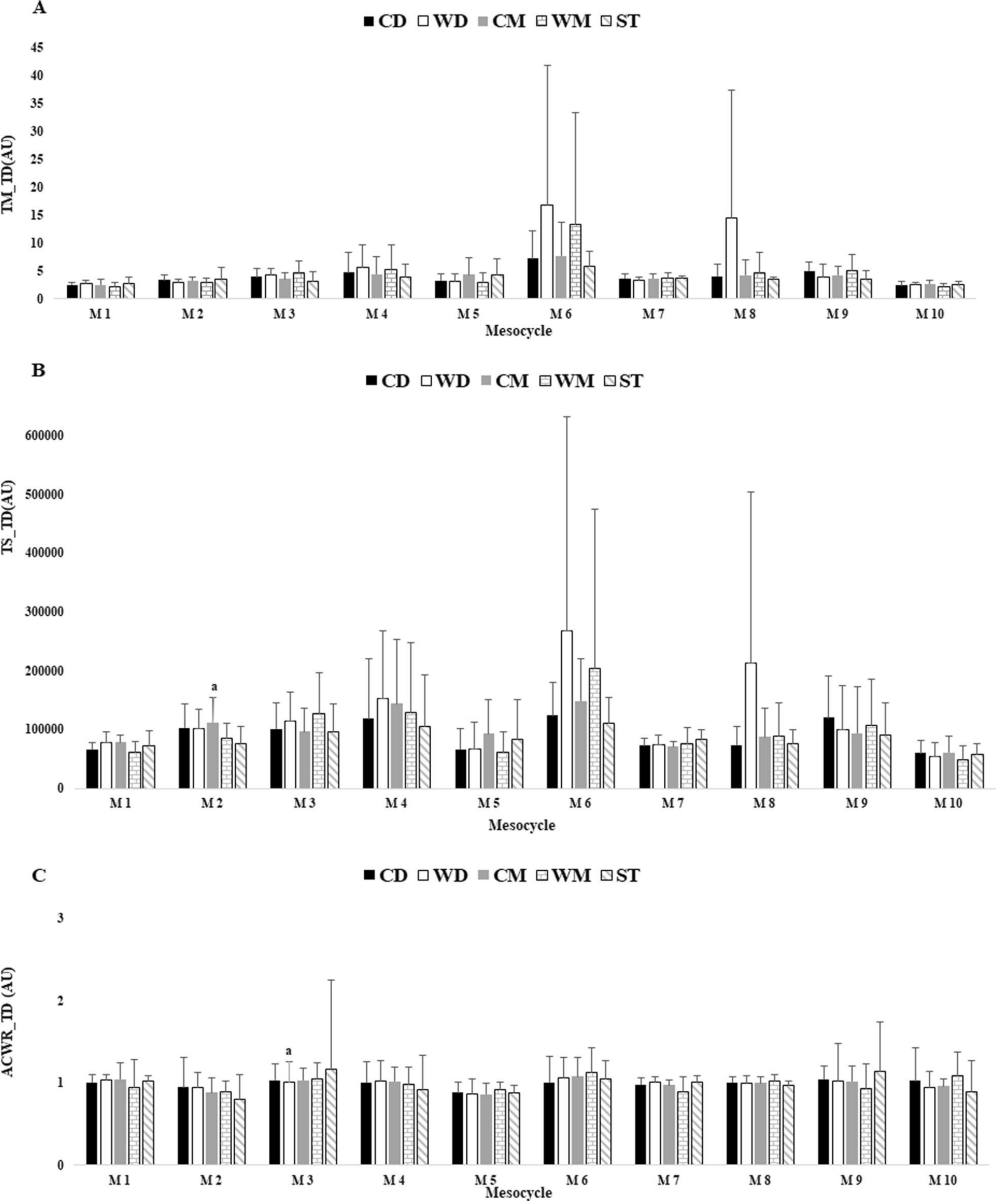
TM, TS and ACWR variations calculated through the TD across 10 mesocycles weeks by player positions. **a** TM_TD; **b** TS_TD; **c** ACWR_TD. M: mesocycle; a: denotes difference from strikers. All, p < 0.05

**Fig. 3:**
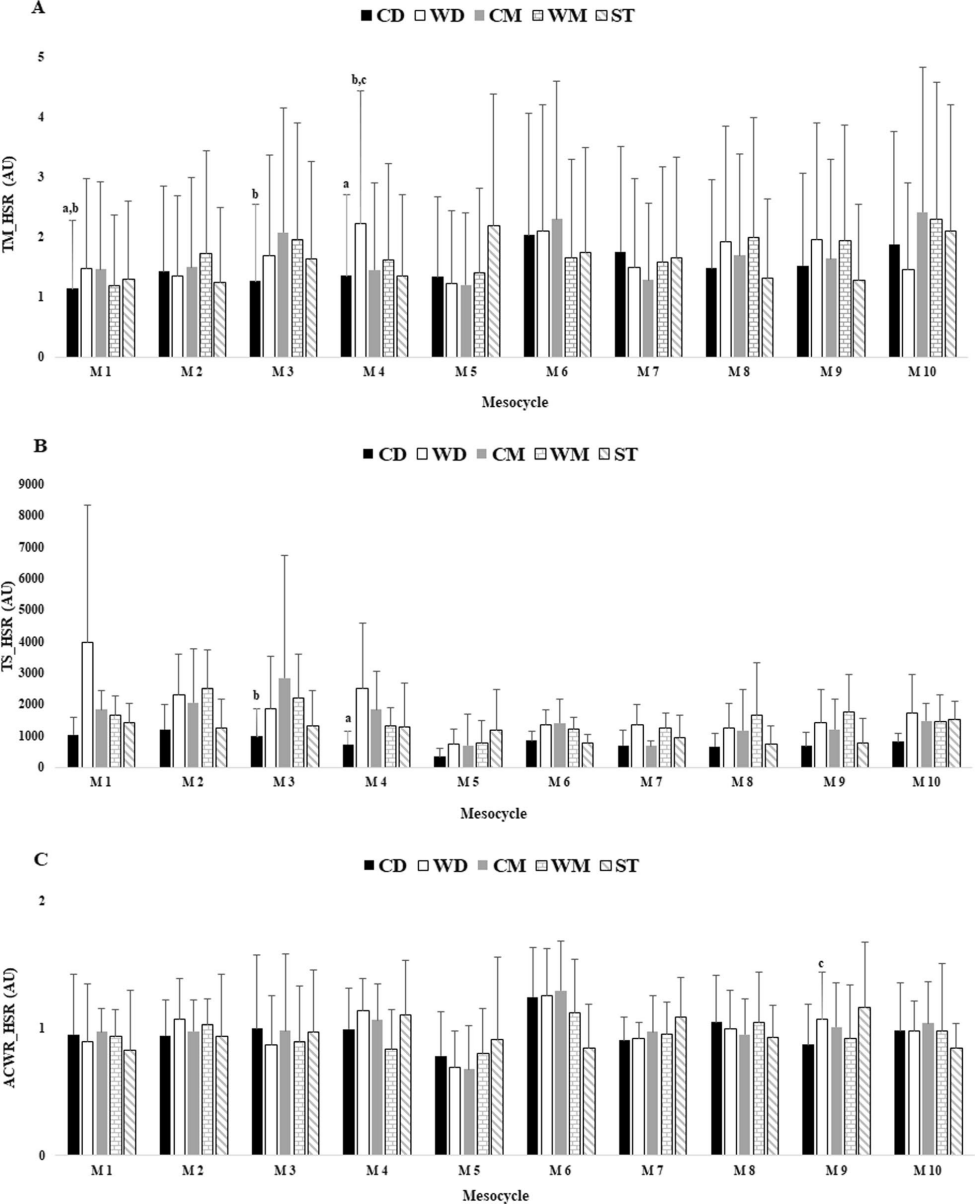
TM, TS and ACWR variations calculated through the HSR across 10 mesocycles weeks by player positions. **a** TM_HSR; **b** TS_HSR; **c** ACWR_HSR. M: mesocycle; a: denotes difference from wide defenders; b: denotes differences from central midfielders; c: denotes differences from strikers. All, p < 0.05

Figures 1, 2 and 3 showed the differences between player positions for TM, TS, and ACWR calculated through the s-RPE, TD and HSR across the in-season.

Overall, Fig. 1a showed that the highest TM_s-RPE_ occurred in M6 for wide midfielders (6.6 Arbitrary Units (AU)) and the lowest value in M5 for central defenders (1.3 AU). There only was one significant difference for TM_s-RPE_ between central defenders vs. strikers (ES = 1.0 [− 0.59; 2.29], moderate effect size) in M2. The highest TS_s-RPE_ occurred in M9 for central midfielders (7673.3 AU) and the lowest value occurred in M5 for central defenders (1120.3 AU). There was significant difference between central defenders vs. central midfielders (ES = − 0.77 [− 2.20; 0.67], moderate effect size), and wide midfielders vs. central midfielders (ES = − 0.17 [− 2.79; 0.45], trivial effect size) in M10. The highest ACWR_s-RPE_ occurred in M6 (1.30 AU) and the lowest value occurred in M5, both by central defenders (0.73 AU). There was significant difference between central midfielders vs. strikers (ES = 0.56 [− 0.97; 2.08], small effect size) in M2 and central defenders vs. strikers (ES = 0.82 [− 0.74; 2.38], moderate effect size) in M6.

Overall, Fig. 2a showed that the highest TM_TD_ occurred in M6 for wide defenders (16.9AU) and the lowest value in M1 for wide midfielders (2.1 AU). There were no differences between player positions. The highest TS_TD_ occurred in M6 for wide defenders (267582.5 AU), and the lowest value occurred in M10 for wide midfielders (47897.9 AU). There was significant difference between central midfielders vs. strikers (ES = 0.79 [− 0.73; 2.33], moderate effect size) in M2. The highest ACWR_TD_ occurred in M3 for strikers (1.17 AU) and the lowest value occurred in M2 for strikers (0.80 AU). There was significant difference between wide defenders vs. strikers (ES = 0.82 [− 1.77; 1.44], moderate effect size) in M3.

Overall, Fig. 3a showed that the highest TM_HSR_ occurred in M10 for central midfielders (2.4 AU) and the lowest value in M1 for central defenders (1.1 AU). There were significant differences for TM_HSR_ between central defenders vs. wide defenders (ES = − 2.26 [− 1.72; 1.28], very large effect size), and central defenders vs. central midfielders (ES = − 0.21 [− 1.60; 1.18], small effect size) in M1; central defenders vs. central midfielders (ES = − 0.47 [− 1.81; 0.99], small effect size) in M3; central defenders vs. wide midfielders (ES = − 0.41 [− 1.93; 1.10], small effect size) and wide defenders vs. central midfielders (ES = 0.36 [− 1.15; 1.87], small effect size) and wide defenders vs. strikers (ES = 0.37 [− 1.24; 1.99], small effect size) in M4. The highest TS_HSR_ occurred in M1 for wide defenders (3972.5 AU) and the lowest value occurred in M5 for central defenders (343.9 AU). There was significant difference between central defenders vs. central midfielders (ES = − 0.57 [− 1.99; 0.84], small effect size) in M3 and central defenders vs. wide defenders (ES = − 1.09 [− 2.69; 0.52], moderate effect size) in M4. The highest ACWR_HSR_ occurred in M6 for central midfielders (1.30 AU) and the lowest value occurred in M5 for central midfielders (0.68 AU). There was significant difference between wide defenders vs. strikers (ES = − 0.17 [− 1.77; 1.43], trivial effect size) in M9.

## Discussion

The purpose of this study was to describe the in-season (ten mesocycles) variations of training monotony (TM), training strain (TS), and acute: chronic workload ratio (ACWR) through session rated perceived exertion (s-RPE), total distance (TD), and high-speed running (HSR) between player positions of an elite soccer team. To the best of the author’s knowledge, this is the first study to analyze such workload measures, including ACWR between player positions. The findings revealed some meaningful variations of workload measures and significant differences between playing positions, only the TM of total distance did not show any significant difference. Contrary to previous study [13], this study found some significant differences between player positions in various mesocycles. Therefore, these results provide new insights for coaches and practitioners about how to plan mesocycles and microcycles according to player positions in a top elite European soccer team.

Regarding the variations of TM, TS, and ACWR through s-RPE (Fig. 1a) between player positions, there were significant differences in all variables in several mesocycles (M). The lowest values for TM (1.3 AU), TS (1120.3 AU), and ACWR (0.73 AU) of s-RPE were observed in M5, for the central defenders (CD). This result may have been a strategy of the coach to prepare the team for the next mesocycle (M6) since it was the mesocycle with the highest number of matches (8 matches). This result is in line with other studies [9, 13, 26], which reported a decrease in acute load due to congested microcycles. In this sense, the highest values for TM (6.6 AU) and ACWR (1.30 AU) of s-RPE were in M6 for wide midfielders (WM) and CD, respectively. In fact, through Table 1, it is possible to see that coach and the technical staff had a special concern for the CD’s, by decreasing the minutes of training in M5 and M6. The differences found between positions through s-RPE confirm the usefulness of this analysis to plan the next mesocycles. For TM of s-RPE there was a significant difference between CD vs. strikers (ST) in M2. For TS of s-RPE there were significant differences between CD vs. central midfielders (CM) and wide defenders (WD) vs. CM in M10. For ACWR of s-RPE, there were significant differences between CM vs. ST in M2 and CD vs. ST in M6. These results once again confirmed that the s-RPE method is a simple, valid, and well-established method [27], due to the fact that it can integrate different types of physiological stimuli referring to the internal load [7]. Moreover, a recent study on elite European players also found no differences on s-RPE considering monotony, strain and ACWR between starters and non-starters which supports the importance of using s-RPE and analysing player positions [16], albeit other study found some differences between starters and non-starters for the same measures in a under 17 soccer team [17]. These findings suggest that results should be carefully interpreted considering specific scenarios from the teams analysed.

A recent study found no significant differences between player positions through TD [13], which is in contrast to our study because we found significant differences for TS of TD for CM vs. ST in M2, and ACWR of TD for WD vs. ST in M3. These differences highlight the importance of the first mesocycles of the season which displayed a relevant focus on the team’s physical preparation. For example, by increasing the volume of training in the present study, there was little variation in acute load for this external measure during the four mesocycles of the in-season, which is in agreement with previous studies [13, 28, 29]. Figure 2B showed that the highest TM of TD values occurred in M6 for all positions, which may be related to the number of matches and training sessions performed in this period. In our opinion, this may not be the most correct load pattern. The “w-shape” fluctuating pattern between mesocycles is the most correct, in order to avoid possible non-traumatic injuries or loss of performance [30, 31]. For instance, a previous study analysed 30 elite soccer players for 45 weeks and it showed a “w-shape” fluctuating pattern between week 1 to week 30 for TM of TD [13]. Interestingly, this reference pattern was easily identified in TS of TD between M4 and M10 for all positions. This variation between measures (TM vs. TS of TD) confirmed a previous study that analysed 36 elite Australian footballers and concluded that there was not always a clear explanation for these discrepancies [32]. To our knowledge this is the first study that included the quantification of the ACWR of TD, by presenting the first reference values through this index. The highest ACWR of TD occurred in M3 for ST (1.17 AU) and the lowest value occurred in M2 for ST (0.80 AU), thus, these values suggest that during the season, the players were within the optimal load zone, because it is suggested that injury likelihood is low when the ACWR is within a range of 0.8–1.3 AU (protection), and high when it exceeds 1.5 AU (risk) [33, 34].

Considering the differences between playing positions and the workload indexes calculated through the HSR, there was meaningfully higher values for CM’s in TM and ACWR, while for WD’s in TS. Our study showed significant differences in workload measures, which was in contrast with a recent previous study [13]. Despite this divergence, differences between playing positions in external load measurements have been documented [35–37], however, these studies only consider three field positions (i.e., defenders, midfielders, and attackers), which reinforces the importance of the present study for coaches and technical teams. In the present study, in TM of HSR, there were several significant differences, namely, in M1 between CD vs. WD and CD vs. CM, in M3 between CD vs. CM, and M4 between CD vs. WM, WD vs. CM, and WD vs. ST. These results suggest that when congested periods begin, the coaching staff should choose to plan the sessions based on other factors (e.g., starters vs. non-starters) [9]. Regarding the pattern of the graphs (Fig. 3), HSR had little TM variation during the season, looking for a “w-shape” fluctuating pattern since the beginning of the in-season, in contrast to the study by Clemente et al. [13] that only achieved this scenario between week 33 and 42. Additionally, our previous study reinforced that this workload measure presented a similar shape when considering starters and non-starters which may help to justify that training load adjustments were applied to to reduce differences according to the player status [16] and player positions.

Finally, concerning TS of HSR, the results showed an increase in the first four mesocycles compared to the other mesocycles for all positions in the study. A study conducted with 26 under-16 elite young soccer players confirmed this result, but only for the CD, WM, and ST [28]. However, our study was in contrast with a previous study conducted with European soccer players [13] that reported a higher TS of HSR during the pre-season, revealing a pattern of 2–5-week mesocycle of lower values, followed by high increases in the following week, throughout the season.

The present study has some limitations that should be acknowledged. First, the size of the sample and only one team were analyzed that although typical in soccer studies may not allow generalizations of the results. This issue is one of the limitations of longitudinal studies over an in-season in professional contexts. Second, pre-season was not analyzed, only the in-season period is evaluated. Third, no measurements were made regarding the injury rate of the players. Fourth, we did not consider other accelerometry measures, such as the number of sprints or/and player load or other objective internal load measure than s-RPE, which could give more information about the quantification of physiological responses to the training sessions. Finally, high-speed running threshold was set ≥ 19 km/h for all players. This approach could be improved in future studies if an individualized velocity was defined for each player or at least for each position.

Despite the limitations, this study was the first, to the best of our knowledge, to describe the in-season variations of TM, TS, and ACWR through s-RPE, TD, and HSR between player positions of an elite soccer team. This study analysed all field positions (except for goalkeepers), unlike some studies that only divide them into three positions. Finally, this study included internal and external load variables, quantified subjectively (s-RPE) and directly (TD and HSR) which could be considered a major strength of this study.

## Conclusions

The results of the present study revealed several differences between player positions over the season. In addition, the results showed the relevance of avoiding isolated peaks of load along the season, especially in congested periods. The most usual pattern of training load distribution in a fluctuating “W-shape curve” along the season. Thus, the present study presents new insights to coaches and technical staff about the variation profiling of TM, TS, and ACWR calculated with internal and external load measures, between player positions during 10 mesocycles.

## Data Availability

The data presented in this study are available on request from the corresponding author.

## Abbreviations

TM: Training monotony
TS: Training strain
ACWR: Acute: chronic workload ratio
s-RPE: Session rating of perceived exertion
HSR: High-speed running
AU: Arbitrary units
BMI: Body mass index
CD: Central defenders
CM: Central midfielders
WD: Wide defenders
WM: Wide midfielders
ST: Strikers
M: Mesocycles
ES: Effect size
